# Lung Cancer Incidence and Mortality in a Nationwide 45-year Prospective Cohort of Persons with Opioid Use Disorder

**DOI:** 10.21203/rs.3.rs-11157942/v1

**Published:** 2026-10-22

**Authors:** Xinyu Wang, Erin Esposito, James J. Goedert, Qiao Zhang, Erin Oxnam, Bridget R. Vaydovsky, Shihui Peng, Andrew W. Bergen, Daniel M. Rosenblum, Stanley H. Weiss

## Abstract

Purpose To examine lung cancer (LC) incidence and mortality in persons with opioid use disorder (OUD) in a national prospective longitudinal cohort. Methods Participants (N = 9895) were recruited from US medication-assisted treatment clinics. LC cases and deaths were ascertained through state cancer registries and the National Death Index-Plus. Covariates came from questionnaires and medical records. We calculated standardized incidence ratios (SIRs) and mortality ratios (SMRs) based on general population statistics; survival was estimated using Kaplan-Meier methods. Results There were 245 LC cases (56.7% distant stage at diagnosis) and 186 LC deaths, double the expected rates (SIR, 2.03, 95% CI: 1.78–2.30; SMR, 2.19, 95% CI: 1.88–2.52). Risks were similar among non-Hispanic Whites (N = 3227) and non-Hispanic Blacks (N = 3943), higher than Hispanics. SIR and SMR showed a dose-response trend with increasing smoking frequency. Among tobacco smokers with < 20 pack-years, the increased risk was similar to other pack-year groups. Risks were higher with HIV (N = 2079, SIR = 3.31, 95% CI: 2.43–4.40), for both prevalent and incident HIV. Median LC-specific survival was 76 months, 26 months, and 5 months, with local, regional, and distant stage at diagnosis, respectively. LC was frequently diagnosed at late stage. Conclusion LC incidence and mortality rates were two-fold elevated. Elevated risks with < 20 pack-years of smoking suggest current LC screening criteria may be too stringent for this population. Integrating LC and tobacco-cessation efforts into drug treatment programs, targeting both providers and patients, may reduce LC morbidity and mortality. Further research with larger, diverse cohorts is needed to confirm these findings.

